# Cardioprotective Antioxidant and Anti-Inflammatory Mechanisms Induced by Intermittent Hypobaric Hypoxia

**DOI:** 10.3390/antiox11061043

**Published:** 2022-05-25

**Authors:** Alejandro González-Candia, Alejandro A. Candia, Adolfo Paz, Fuad Mobarec, Rodrigo Urbina-Varela, Andrea del Campo, Emilio A. Herrera, Rodrigo L. Castillo

**Affiliations:** 1 Institute of Health Sciences, University of O’Higgins, Rancagua 2820000, Chile; alejandro.gonzalez@uoh.cl; 2 Laboratory of Vascular Function & Reactivity, Pathophysiology Program, ICBM, Faculty of Medicine, Universidad de Chile, Santiago 7500922, Chile; alejandrocandiah@uchile.cl (A.A.C.); adolfopazf@gmail.com (A.P.); fuadml@yahoo.es (F.M.); 3 Department for the Woman and Newborn Health Promotion, Universidad de Chile, Santiago 7500922, Chile; 4 Laboratorio de Fisiología y Bioenergética Celular, Facultad de Química y de Farmacia, Pontificia Universidad Católica de Chile, Santiago 7820436, Chile; rodrigo.urbina.83@gmail.com (R.U.-V.); andrea.delcampo@uc.cl (A.d.C.); 5 International Center for Andean Studies (INCAS), University of Chile, Putre 1070000, Chile; 6 Departamento de Medicina Interna Oriente, Facultad de Medicina, Universidad de Chile, Santiago 7500922, Chile; 7 Unidad de Paciente Crítico, Hospital del Salvador, Santiago 7500922, Chile

**Keywords:** intermittent hypoxia, NLRP3 inflammasome, antioxidant defenses, NF-kappaB, cardioprotection

## Abstract

More than 80 million people live and work (in a chronic or intermittent form) above 2500 masl, and 35 million live in the Andean Mountains. Furthermore, in Chile, it is estimated that 100,000 people work in high-altitude shifts, where stays in the lowlands are interspersed with working visits in the highlands. Acute exposure to high altitude has been shown to induce oxidative stress in healthy human lowlanders due to increased free radical formation and decreased antioxidant capacity. However, intermittent hypoxia (IH) induces preconditioning in animal models, generating cardioprotection. Here, we aim to describe the responses of a cardiac function to four cycles of intermittent hypobaric hypoxia (IHH) in a rat model. The twelve adult Wistar rats were randomly divided into two equal groups, a four-cycle of IHH and a normobaric hypoxic control. Intermittent hypoxia was induced in a hypobaric chamber in four continuous cycles (1 cycle = 4 days of hypoxia + 4 days of normoxia), reaching a barometric pressure equivalent to 4600 m of altitude (428 Torr). At the end of the fourth cycle, cardiac structural and functional variables were also determined by echocardiography; furthermore, cardiac oxidative stress biomarkers (4-Hydroxynonenal, HNE; nitrotyrosine, NT), antioxidant enzymes, and NLRP3 inflammasome panel expression are also determined. Our results show a higher ejection and a shortening fraction of the left ventricle function by the end of the fourth cycle. Furthermore, cardiac tissue presented a decreased expression of antioxidant proteins. However, a decrease in IL-1β, TNF-αn, and oxidative stress markers is observed in IHH compared to normobaric hypoxic controls. Non-significant differences were found in protein levels of NLRP3 and caspase-1. IHH exposure determines structural and functional heart changes. These findings suggest that initial states of IHH are beneficial for cardiovascular function and protection.

## 1. Introduction

Several studies have reported that intermittent hypoxic training can provide evident measurable protection in pathophysiological states or enable improvements in developed sports-related performances [1,2]. The protective effects of intermittent hypoxia (IH) can be explained by the activation and propagation of adaptive responses induced by the IH stimulus, usually through a process that has been generally termed preconditioning. Thus, short exposures to mild IH episodes can protect specific cells, tissues, or organs against more severe hypoxic and ischemic insults [3,4]. However, the level of preconditioning and hypoxic intensity and their cardiovascular benefits are still under debate.

Animals subjected to various paradigms of acute IH become more resistant to the lethal injury induced by subsequent exposures to severe hypoxic insults [5,6,7]. For instance, when compared to controls, mice treated with brief episodes of IH exposure (8% O_2_ × 10 min/21% O_2_ × 10 min, 1 h) showed attenuated cellular and tissue injury in crucial organs, such as the lungs, heart, and brain, relative to extended hypoxia [8]. Furthermore, myocardium from mice exposed to a similar IH pattern (6% O_2_ × 6 min/21% O_2_ × 6 min, 1 h) or from rats treated with a higher frequency of IH (10% O_2_ × 40 s/21% O _2_× 20 s, for 4 h) were protected against ischemia-induced infarction [9]. Such IH-induced cardioprotection seems to rely on the activation of similar pathways to those described in models of cardiac ischemic preconditioning, such as increased antioxidant cellular response and induction of anti-inflammatory pathways [8]. These effects are different from those described after the induction of myocardial damage by normobaric hypoxia, such as obstructive sleep apnea, where oxidative stress determines pathological remodeling and impairment in ventricular function [10]. Previously, our group demonstrated that simulating 4 cycles of acute intermittent hypobaric hypoxia (IHH) (428 × 750 Torr, 4 days) induces functional improvement of left ventricular contractility associated with a higher antioxidant enzymatic expression and a reduction of lipid peroxidation products, establishing cardio-protection [4,7,11]. However, the intracellular signaling mechanisms that determine the crosstalk between anti-inflammatory and antioxidant effects are not well characterized. 

The NLRP3 inflammasome may contribute to cardiac damage during hypoxic exposure and reperfusion injury [12]. Inflammasomes are high molecular weight protein complexes in the cytosol of immune and other cells, such as hepatocytes and cardiomyocytes, which play a critical role in the innate immune system in response to cellular stress [13]. The NLRP3 inflammasome is formed through the interaction of a core of intracellular proteins identified as NLRP3 (for a nucleotide-binding domain, leucine-rich containing family, pyrin domain-containing-3), bipartite adaptor protein ASC (an apoptosis-associated speck-like protein containing a caspase recruitment domain or CARD), and effector protein procaspase-1 [14]. Furthermore, the NLRP3 inflammasome is the best-understood inflammasome during chronic diseases and is known to mediate the maturation (activation) of caspase-1 from pro-caspase-1, causing the maturation and release of cytokines (e.g., interleukin-1 and interleukin-18 to IL-1β and IL-18, respectively) and potentially leading to a form of inflammatory programmed cell death called pyroptosis [15]. Preclinical models have shown that the NLRP3 components are expressed in cardiomyocytes and cardiac fibroblasts, and recent studies have identified the NLRP3 inflammasome as a critical nodal point in the pathogenesis of cardiomyopathies and hypoxic injury, which may create an opportunity for the development of new therapeutic agents [16].

Previous studies targeting the inhibition of several inflammatory pathways in acute cardiac hypoxic injury are inconclusive [17,18]. This may happen because inflammation is a double-edged sword, detrimental when hyperactive but beneficial at lower activity, with activity critically dependent on the time of reperfusion and cellular location [19,20]. Therefore, it is reasonable that this also applies to the NLRP3 inflammasome, although current literature has mainly focused on its detrimental effects in acute cardiac hypoxia but not in IHH exposure [21]. The aim of this study was to determine antioxidant and anti-inflammatory cardiac tissue changes in rats subjected to IHH. For this purpose, we assessed cardiac functional and structural parameters, NLRP3 content, and pro-inflammatory cytokines and oxidative markers in a rat model of IHH. 

## 2. Materials and Methods

### 2.1. Animals

All animal care, maintenance, and procedures were approved by the Bioethics Committee of the Faculty of Medicine, University of Chile (CBA 0865 FMUCH), and were carried out in accordance with Guidelines for the Care and Use of Laboratory Animals. The experiment protocols were performed on 8-week-old Wistar Kyoto male rats. Animals were housed in standard conditions in a temperature and light-controlled room (20–24 °C; 12 h of light, 12 h of dark). Twelve rats were randomly divided into the following two equal groups: a control group maintained in normobaric normoxia (NN, 750 Torr, *n* = 6) at near-sea level; an intermittent hypobaric hypoxic group (IHH, *n* = 6), exposed to one shift of hypobaric hypoxia (428 Torr, 4 days) followed by one shift of normoxia (750 Torr, 4 days). Completion of 2 shifts was considered 1 cycle. IHH group was exposed to 4 cycles. All animals were maintained in a homemade automated controlled-pressure chamber, and the chamber was programmed for specific pressure exposure. 

### 2.2. Echocardiography

Echocardiographic examinations were performed by the end of the 4 cycles in both experimental groups in sedated animals (Ketamine/xylazine 60:10 mg kg; Sonosite 180 Plus and a 10 MHz linear transducer). To determine the cardiac morphology and function, we assessed the ejection fraction (EF), shortening fraction (SF), heart rate (HR), diastolic (LVDD), and systolic (LVSD) diameters of the left ventricle, and left atrium (LADD) [7].

At the end of the experimental protocol, the animals were euthanized with an anesthetic overdose (Sodium Thiopentone 150 mg·kg^−1^ IP), and their hearts were excised for histology and molecular biology assays. 

### 2.3. Heart Collection

The hearts were removed, weighed, and the ventricles were cross-sectioned equidistantly between the apex and the atrioventricular limit. The inferior portion was stored in 4% paraformaldehyde during 24 h for histological analysis. The other half was frozen and stored at −80 °C for biochemical and molecular biology analyses. 

### 2.4. Cardiac Histology

Fixed samples of the heart were embedded in paraffin and cut into 5 μm thick slides. Hematoxylin-Eosin staining was performed for cardiomyocyte density in images captured at 100× and 400× with a digital camera coupled to a microscope (Olympus BX-41). Briefly, we determined the left and right ventricular luminal areas, the interventricular septum (IS) thickness, and both ventricular-free walls. In addition, for each ventricle wall and IS, we determined the myocardial cellular density as the number of nuclei/area. The analysis of the microphotographs was performed with the software Image Pro-Plus 6.2 (Media Cybernetics Inc., Rockville, MD, USA) [22].

### 2.5. Protein Levels in the Cardiac Tissue

Protein levels of TNF-α, NF-κB, NLRP3, IL-1β, IL-6, IL-8, Nrf2, pro-caspase and cleaved caspase-1, CAT, GPx-1, GPx-2, GPx-4, SOD-1, SOD-2, SOD-3, and αβ-tubulin were determined in total cardiac tissue. The cells were lysed in RIPA buffer (Thermo Scientific, Rockford, IL, USA). The Bradford protein assay determined the concentration of complete protein. Protein samples were separated by SDS PAGE and transferred to nitrocellulose membranes. The membranes were incubated with primary antibodies (anti- TNF-α, Abcam, ab6671; anti- NF-κB, Abcam, ab16502; anti-NLRP3, R&D system, MAB7578; anti- IL-1β, Santa Cruz Biotechnology, sc-12742; anti-IL-6, Santa Cruz Biotechnology, sc-57315; anti- IL-8, Santa Cruz Biotechnology, sc-376750; anti- Nrf2, Abcam, ab137550; anti-caspase-1, Abcam, ab207802; anti-CAT, Abcam, ab1877; anti-GPx-1, Abcam, ab22604; anti-GPx-2, Santa Cruz Biotechnology, sc-133160; anti-GPx-4, Santa Cruz Biotechnology, sc-166570, anti-SOD1, Santa Cruz Biotechnology, sc-17767; anti-SOD2, Millipore, 06-984; anti-SOD3, Santa Cruz Biotechnology, sc-376948; anti-4-HNE, Abcam, ab46545; anti-NT, Millipore 05-233 and anti-α/β-tubulin, cell signaling, # 2148, respectively), washed and incubated with secondary antibody anti-rabbit or mouse according to the manufacturer’s instructions. All proteins were normalized to αβ-tubulin levels. The signals obtained on immunoblot determinations were scanned and quantified by densitometric analysis by a chemoluminescence detection device (Odyssey Imaging System, Li-Cor Biosciences, Lincoln, NE, USA). 

### 2.6. Statistical Analyses

All data were expressed as mean ± SEM. Shapiro–Wilk test was used to evaluate the normality of the data. Accordingly, cardiovascular data, morphometry, and molecular biology assessment were compared statistically by an unpaired *t*-test. Significant differences were accepted when *p* ≤ 0.05 (Prism 9.0, GraphPad Software, San Diego, CA, USA) [23]. 

## 3. Results

### 3.1. Effects of IHH on In Vivo Cardiac Morphometry and Function

**Figure 1:**
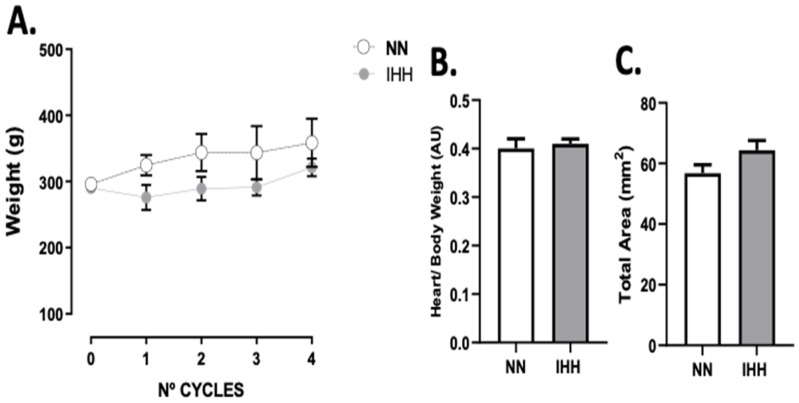
Body and heart weight. The animals’ body weight was measured at the beginning of each experimental cycle (**A**). In addition, heart/body weight (**B**) was measured at euthanasia., total area (**C**) are shown. Groups are normobaric normoxia (NN, *n* = 6) and intermittent hypobaric hypoxia (IHH, *n* = 6). Data are expressed in mean ± SEM.

The animals’ body weight was measured at the beginning of each experimental cycle, with no differences between groups (Figure 1A). Furthermore, the heart/body weight ratio (Figure 1B), and total area (Figure 1C) at the end of the 4 cycles were similar in both groups. 

**Figure 2:**
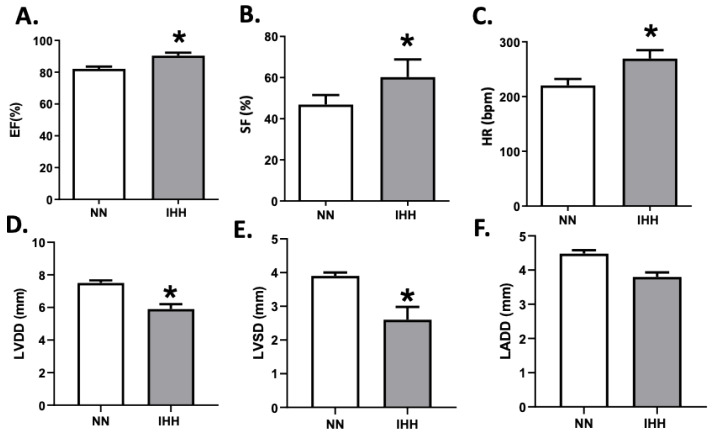
Echocardiographic parameters. The left cardiac function was evaluated by ultrasound echocardiography through the ejection fraction (EF%, (**A**)), shortening fraction (SF%, (**B**)), heart rate (HR, (**C**)), left ventricular diastolic diameters (LVDD, (**D**)), the systolic diameter of the left ventricle (LVSD, (**E**)) and left atrial diameter (LADD, (**F**). Groups are normobaric normoxia (NN, *n* = 6) and intermittent hypobaric hypoxia (IHH, *n* = 6). Data are expressed in mean ± SEM. Significant differences (*p* ≤ 0.05): * vs. NN.

**Figure 3:**
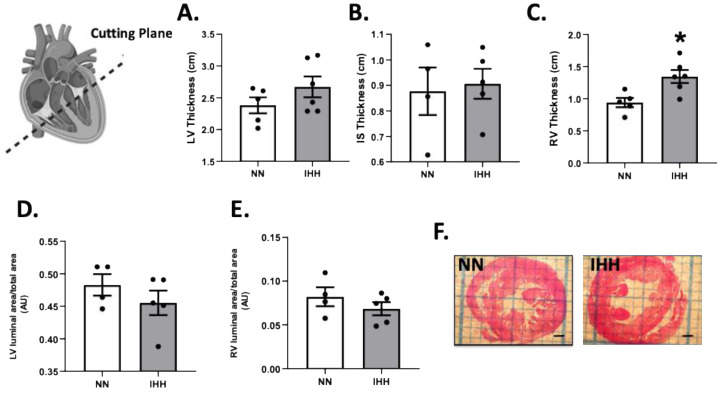
Cardiac morphometry. Left ventricle (LV) thickness (**A**), interventricular septum (IS) thickness (**B**), right ventricle (RV) thickness (**C**), LV luminal area/total area (**D**), and RV luminal area/total (**E**); (**F**) cutting plane depiction. Groups are normobaric normoxia (NN, *n* = 6) and intermittent hypobaric hypoxia (IHH, *n* = 6). Data are expressed in mean ± SEM. * statistically significant vs. NN.

During the experimental protocol, rats in the IHH group showed an increase in EF, SF, and HR compared to the NN group (Figure 2A–C). On the other hand, IHH significantly decreased LVDD and LVSD without changes in LADD (Figure 2D–F). Furthermore, LV thickness, IS thickness, LV luminal area/total area, and RV luminal area/total area remained similar in IHH and NN groups (Figure 3A,B,D,E). However, RV thickness (3C) is higher in the IHH group, as a measure of probable ventricular remodeling. 

### 3.2. Cardiac NLRP3 Inflammasome and Cytokines Protein Levels

**Figure 4:**
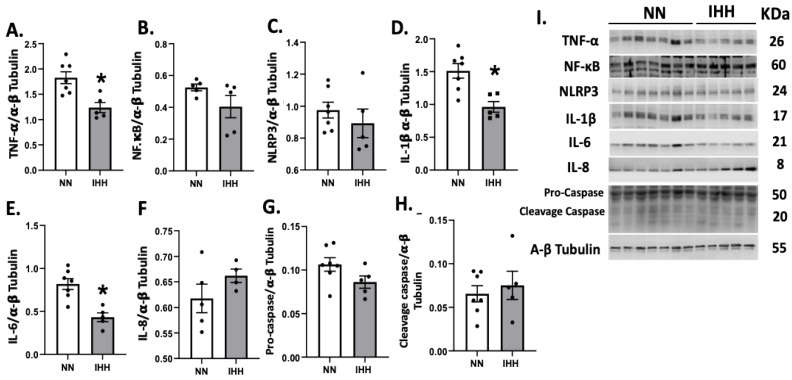
Cardiac levels of inflammatory-related proteins. Cardiac levels of TNF-α (**A**), NF-KappaB p65 subunit (**B**), NLRP3 (**C**), IL-1β (**D**), IL-6 (**E**), IL-8 (**F**), Procaspase-1 (**G**), and cleaved-caspase 1 (**H**). (**I**), corresponds to blot bands and standard weight. Groups are normobaric normoxia (NN, *n* = 6) and intermittent hypobaric hypoxia (IHH, *n* = 6). Data are expressed in mean ± SEM. Significant differences (*p* ≤ 0.05): * vs. NN.

TNF-α was significantly reduced after the exposure to IHH compared to controls (Figure 4A); however, NF-κB and NLRP3 remained similar in both groups (Figure 4B,C). Interestingly, IL-1β and IL-6 decreased in IHH (Figure 4D,E), whereas there were no changes in IL-8, pro-casapse-1, and caspase-1 (Figure 4F–H). 

### 3.3. Cardiac Redox State

**Figure 5:**
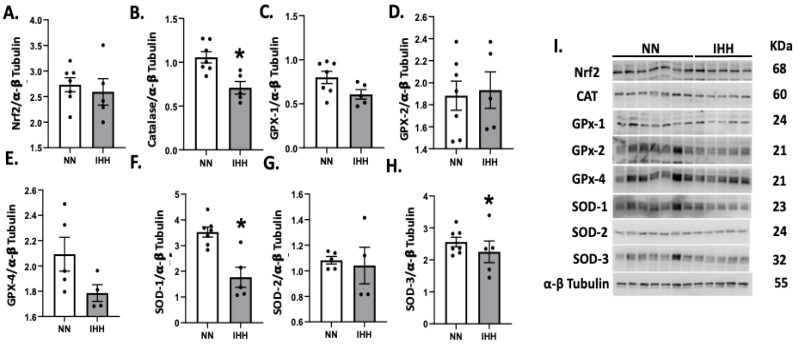
Cardiac levels of antioxidant-related proteins. The cardiac protein levels of Nrf2 (**A**) and the antioxidants enzymes, Catalase (CAT, (**B**)), Glutathione peroxidase-1 (GPX-1, (**C**)), Glutathione peroxidase-2 (GPX-2, (**D**)), Glutathione peroxidase-4 (GPX-4, (**E**)), and Superoxide Dismutase (SOD-1, 2, and 3 (**F**–**H**)). (**I**), corresponds to blot bands and standard weight. Groups are normobaric normoxia (NN, *n* = 6) and intermittent hypobaric hypoxia (IHH, *n* = 6). Data are expressed in mean ± SEM. Significant differences (*p* ≤ 0.05): * vs. NN.

**Figure 6:**
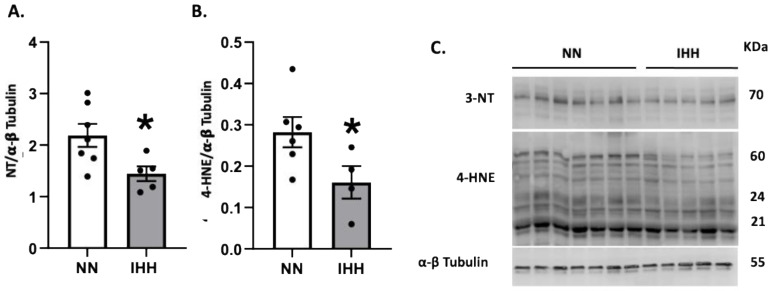
Cardiac oxidative stress. Quantification of cardiac protein levels of 3-Nitrotyrosine (3-NT) (**A**) and 4-hydroxynonenal (4-HNE) (**B**) as oxidative stress markers. Western blot bands are shown (**C**). Groups are normobaric normoxia (NN, *n* = 6) and intermittent hypobaric hypoxia (IHH, *n* = 6). Data are expressed in mean ± SEM. Significant differences (*p* ≤ 0.05): * vs. NN.

Protein levels of Nrf2 were similar in both groups (Figure 5A), whereas the levels of CAT and SOD 1 and 3 were decreased in IHH compared to NN (Figure 5B,F,H). In contrast, GPX-1, GPX-2, GPX-4, and SOD-2 and SOD-3, (Figure 5C–H) maintained similar protein levels in both groups. Finally, cardiac oxidative stress markers such as 3-NT and 4HNE were significantly reduced in IHH compared to NN (Figure 6A,B). 

The Figure S1 show the Western blot full bands. 

## 4. Discussion

This study showed that IHH induces a preconditioning effect in cardiac tissue mediated by decreased oxidative stress markers. We conducted diverse experimental approaches showing that IHH improves myocardial ventricular function without affecting the cardiac structure. In addition, the functional improvement was associated with a decreased antioxidant and anti-inflammatory-related protein induction. Our findings are novel, considering that there are few studies regarding the IHH animal model. 

For this experimental protocol design, we used a hypobaric chamber to study the pathophysiological mechanisms induced by IHH as a translational form to show the cardioprotection clinically observed in high-altitude workers or intermittent residents above 2500 m above sea level (masl) [4,7]. Recently, experimental evidence has shown in animal models of injury due to cerebral and myocardial hypoxia that the high intensity of IHH (over simulated 5000 masl) and short-term exposure time (6–12 h/day per 7–10 days) induces ischemic tolerance in target organs [8]. The cardioprotective effect is evidenced by a smaller infarct size or a diminution in the hypoxic risk zone in rats subjected to preconditioning by the IHH protocol [4,24]. However, the functional changes in parameters associated with cardiac contractility are not well characterized.

Furthermore, the effects on systolic function and other dynamic measurements could have contradictory behavior [25]. Therefore, we expected to detect changes in ventricular function as an expression of cardioprotective mechanisms triggered by IHH exposure. In this view, our results show an improvement in left ventricular function independent of cardiac morphometric parameters. Several mechanisms have been involved in the IHH-induced cardioprotective effects, including activation of hypoxia-responsive genes, amelioration of coronary circulation, activation of protein kinase C, the balance of Ca^2+^ handling activity, and inhibition of mitochondrial permeability transition pores (mPTP) opening [26,27]. However, the molecular mechanisms of IHH and the totality of its effects on cardiac function are still unclear. 

The pathophysiology of IHH exposure involves the generation of different levels of ROS depending on the intensity of hypoxia. For this reason, reactive oxygen species can be both harmful and protective [28,29]. It has been shown that several intracellular sources, including mitochondria, NADPH oxidase, xanthine oxidase, and uncoupled nitric oxide synthase, can generate ROS [8,30]. The effects associated with ROS damage can be seen in increased oxidative damage to cellular structures. In this context, markers of oxidative damage in proteins and lipids are the most sensitive and reproducible in in vivo models of IHH [31,32]. Our results show a reduction in cardiac oxidative stress markers such as 3-NT and 4HNE, indicating less protein and lipid damage, consistent with an improved ventricular function. Interestingly, these results were not associated with reinforcing the antioxidant defense system. One way to explain this event would be a positive modulation of cardiac pro-oxidant sources in the myocardium of rats subjected to IHH, such as the increased activity of NADPH oxidase described by Aguilar et al. 2018 [7]. In spite of this, we previously observed that there is a decrease in oxidative stress associated with an increase in enzymatic antioxidant capacity in intermittent hypobaria [4]. The erythroid 2 nuclear factor (Nrf2) [33] actions stimulate the transcription of antioxidant enzymes, thus increasing the antioxidant capacity (enzymatic and non-enzymatic) in a compensatory way to a ROS increase [34]. However, as in our model, Nrf2 is not modified by IHH, we proposed an alternative regulation of oxidative balance, for instance by a decrease in pro-oxidant sources’ expression or activity [35]. Furthermore, reducing ROS production by antioxidants abolished the IHH-induced prevention of cell death in a model of ischemia/reperfusion (I/R) in cardiomyocytes [36,37]. Complementary, Estrada et al. found that treating dogs with IH for 20 days induces significant cardioprotection against I/R, manifested by a 90% reduction in left ventricular infarct size. However, the cardioprotective effects of IH are abolished by pre-treatment with the antioxidant N-acetylcysteine, demonstrating that ROS are critical in regulating cardioprotection caused by IH [27,38]. In cells, a large amount of ROS induces severe oxidative stress, which causes damage to DNA, lipids, and proteins, thereby contributing to many different disease developments, including cardiovascular diseases [39]. However, it has also been demonstrated that ROS can serve as redox signaling molecules in physiological conditions. In this regard, the nuclear factor E2-related factor 2 (Nrf2)-small Maf heterodimer binds to the antioxidant-responsive element (ARE), resulting in the upregulation of antioxidant genes, including SOD and GPx. In addition, Kelch-like ECH-associated protein-1 (Keap1) is a redox sensor associated with NFE2-like 2 (Nrf2). ROS oxidation of cysteines in Keap1 leads to Nrf2 release and translocation into the nucleus [38,39]. Our data revealed that IHH did not promote changes in Nrf2 and CAT/GPX protein levels. However, it has been described that lethal ROS generation upregulates the expression of Cu/Zn SOD and MnSOD proteins and increases catalase and GPx activity [36]. Previously, in a cell IHH model, it has been observed that the mRNA levels of CAT and GPx did not change in a cell model of IHH, whereas the enzymatic activity of CAT and GPx increased. In addition, CAT mRNA half-life varies from several minutes to several hours [36,40]. In a cellular model of chronic IHH, mRNA was degraded but protein expression and activity were still increased [36,40,41]. However, the mechanisms underlying ROS increase and modulation of antioxidant enzyme expression in our model of IHH still require further investigation; for example, we could explore the antioxidant-specific activity and/or the presence of post-translational modifications. 

The cardiac structural and functional data presented in this manuscript are consistent with Aguilar et al.’s 2018 paper [7], reflecting the reproducibility of the IHH model. However, in this paper we assessed the expression of different isoforms of antioxidant enzymes, giving a slightly different result in GPX (decreased GPX4 vs. increased total GPx). These unexpected differences may be explained by the methodological modification and/or could account for the compartmentalization of the oxidative balance [42].

Regarding anti-inflammatory mechanisms induced by IHH and preconditioning cardiac effects, previously, our group described that the cardioprotective response induced by IHH was associated with a lower pro-inflammatory effect mediated by a lower activation of NF-κB at the cardiac level, with the same number of infiltrations of polymorphonuclear neutrophils [4,11]. Therefore, local anti-inflammatory mechanisms could contribute to the cardioprotective effects of IHH at a tissue level and be manifested by a decrease in infarct size [4,43]. Moreover, at a clinical level, IHH is associated with low-grade systemic inflammation characterized by the presence of circulating markers of inflammation such as C-reactive protein, cytokines (e.g., IL-6, TNF-α), and adhesion molecules (e.g., intercellular adhesion molecule-1, vascular cell adhesion molecule-1, selectins). The cytokines and adhesion molecules correlate with the severity of tissue damage in the heart and brain [44,45]. Indeed, many studies have demonstrated systemic and localized inflammation, characterized by increased pro-inflammatory cytokines and chemokines [46], and macrophage recruitment [46]. Moreover, no anti-inflammatory treatment has been shown to improve cardiovascular and metabolic disorders in patients with Obstructive Sleep Apnea (normobaric intermittent hypoxia) [47,48]. Therefore, those molecular targets linking the pro-inflammatory and pro-oxidant responses to IHH models would be attractive in designing new pharmacological strategies to enhance preconditioning effects.

Regarding anti-inflammatory cytokines, our results show that IL-6 and TNF-a have lower levels in the IHH protocol compared to NN rats (Figure 4A,E), as a potential cardioprotective mechanism triggered by IHH. These molecular effects have been previously described by our group in the same animal model, for example determining low levels of NF-κB following IHH induction in ex vivo cardiac I/R injury [4]. TNF-α exists in membrane-bound or cytosolic forms and exerts its actions by binding to cell membrane TNFR1 or TNFR2 receptors [49]. TNF-α has been shown to mediate several adverse effects on heart function and structure, namely, negative inotropic actions due to the disruption of calcium homeostasis, and upregulation of other inflammatory molecules, including induction of inducible NO synthase, enhancement of oxidative stress. For another hand, IL-6 has recently been described in the heart. The IL-6 receptor can be cleaved by proteases, originating a soluble form that can bind IL-6 and initiate signaling in cells that do not express this receptor, thus increasing the complexity of IL-6 cellular effects [50]. Regarding the heart, IL-6 has been shown to exert negative inotropic effects and to promote hypertrophy and fibrosis, contributing to increased myocardial stiffness. For these reasons, its role as an anti-inflammatory and eventually cardioprotective cytokine could be discussed.

Cardiomyocytes and cardiac fibroblasts also play a significant role in controlling pathogen-associated molecular patterns leading to injury and cardiovascular dysfunction via Toll-like receptors and nucleotide-binding domain and (NOD)-like receptors (NLRs) [51,52]. The NLRP3 inflammasome is formed through the interaction of a core of intracellular proteins identified as NLRP3 (for a nucleotide-binding domain, leucine-rich containing family, pyrin domain-containing-3), bipartite adaptor protein ASC (an apoptosis-associated speck-like protein containing a caspase recruitment domain or CARD), and effector protein procaspase-1 [14]. Recent studies have identified the NLRP3 inflammasome as a key nodal point in the pathogenesis of cardiomyopathies and heart dysfunction, which may create an opportunity to develop new therapeutic agents [16]. An enhanced inflammatory response is frequently associated with heart dysfunction development, and increased levels of circulating IL-1β and IL-18 are positively correlated with progression of contractile dysfunction and pro-arrhythmogenic effects and the induction of electrical and structural remodeling in the overload rat model [53]. Recently, it has been shown that the activity of the NLRP3 inflammasome is increased in cardiomyocytes from patients with chronic IH [52,53,54]. However, the study suggests that oxidative stress leads to inflammation by mechanisms other than activation of the NLRP3 inflammasome in chronic IH patients. Furthermore, chronic IH and body mass index (BMI) influenced the serum concentration of inflammatory mediators [55,56,57]. Recently, inflammasome NLRP3 has been described as a mediator of cardiovascular preconditioning [19] and can be considered protective for the organism. From that perspective, it is anticipated that NLRP3 deletion or inhibition would be detrimental to the overall well-being of the organism but also likely to the condition of cardiac I/R injury. The published preclinical literature on the inflammasome regarding the cardiac I/R injury field reports positive effects of NLRP3 inflammasome deletion or inhibition [19]. Although the positive bias to present positive results in preclinical studies may contribute to this, the question is begged whether there are any beneficial (or neutral) effects of the NLRP3 inflammasome in the context of cardiac infarction [56,57]. In our results, we did not show differences in the amount of NLRP3, nor the activation of caspase-1. Probably the prolonged hypoxic exposure per cycle (4 days) may trigger pro-inflammatory mechanisms even in the presence of a preconditioning effect on its ventricular function. Indeed, the isolated NLRP3-/- hearts contained significantly less cytokine IL-6, with a non-significant trend of decreased IL-1 [57]. Moreover, when hearts were isolated from NLRP3-deficient mice, perfused, and subjected to global I/R, a marked improvement in cardiac function and a reduction of hypoxic damage were found compared with wild-type hearts. Moreover, other components of this pathway, such as ASC, IL-6, and STAT3, have a protective role in ischemic preconditioning [58]. Still, these effects in IHH models and intracellular signaling are not well characterized. Therefore, there is sufficient evidence for a potential role of NLRP3 in cardioprotection; however, it remains to be better characterized whether the in vivo anti-inflammatory and antioxidant response is associated with this molecular pathway or is an epiphenomenon.

## 5. Conclusions

Our translational model representing high-altitude shifts such as those seen in working schedules in mining companies and astronomical observatories, among other activities, aims to understand the cardiovascular pathophysiology of such workers. This study gives a better overview of the cardiac functional and structural responses to IHH, serving as a baseline for future biomedical analyses and mechanistic studies. Our findings suggest that IHH improves cardiac function. However, complementary studies are necessary to fully understand the pathophysiological effects of high-altitude shifts, mainly with respect to anti-inflammatory pathways. This is particularly relevant in countries where the economy depends on high-altitude activities. However, as in other cardiac pathologies that depend on the modulation of antioxidant pathways, the NLRP3 inflammasome could be a pharmacological target to be explored as a new paradigm for cardioprotective therapy.

## Data Availability

Data is contained within the article or supplementary material.

## Supplementary Materials

The following supporting information can be downloaded at: https://www.mdpi.com/article/10.3390/antiox11061043/s1, Figure S1: Expression levels of NLRP3 inflammasome and 1L-1β.
