# Supplementary material for: Cardioprotective Antioxidant and Anti-Inflammatory Mechanisms Induced by Intermittent Hypobaric Hypoxia
Source: Antioxidants (Basel). 2022 May 25;11(6):1043. doi: 10.3390/antiox11061043 (PMC9220055; doi:10.3390/antiox11061043)
Supplement: Supplementary file 1 [file antioxidants-11-01043-s001.zip › antioxidants-1708559-supplementary.pdf]

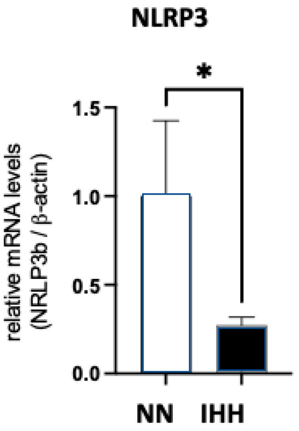

Figure S1. Expression levels of NLRP3 inflammasome and IL-1β. The cardiac mRNA relative levels of NLRP3 inflammasome (Figure S1) Groups are NN: normobaric normoxia (n=6) and IHH: intermittent hypobaric hypoxia (N=6). Data are expressed in mean ± SEM. Significant differences ($p \leq 0.05$): * vs. N.
